# Association of HLA-A*11:01, -A*24:02, and -B*18:01 with Prostate Cancer Risk: A Case-Control Study

**DOI:** 10.3390/ijms242015398

**Published:** 2023-10-20

**Authors:** Maria Antonietta Manca, Elena Rita Simula, Davide Cossu, Tatiana Solinas, Massimo Madonia, Roberto Cusano, Leonardo Antonio Sechi

**Affiliations:** 1 Dipartimento di Scienze Biomediche, Università di Sassari, 07100 Sassari, Italy; simulaelena@gmail.com (E.R.S.); m.anto.manca@gmail.com (M.A.M.); dcossu@uniss.it (D.C.); 2 Dipartimento di Medicina, Chirurgia e Farmacia, Università di Sassari, 07100 Sassari, Italy; tatiana.solinas@tiscali.it (T.S.); madonia@uniss.it (M.M.); 3 Struttura Complessa di Urologia, Azienda Ospedaliera Universitaria, 07100 Sassari, Italy; 4 CRS4, Pula, 09100 Cagliari, Italy; robycuso@crs4.it; 5 Struttura Complessa di Microbiologia e Virologia, Azienda Ospedaliera Universitaria, 07100 Sassari, Italy

**Keywords:** HLA-A*11:01, HLA-A*24:02, HLA-B*18:01, prostate cancer

## Abstract

The major histocompatibility complex (MHC) loci, the most polymorphic regions within the human genome, encode protein complexes responsible for antigen presentation and CD4+ and CD8+ cell activation. In prostate cancer (PCa), the second most diagnosed cancer in the male population, MHC loci undergo significant changes in their expression patterns, which affect the ability of the immune system to attack and eliminate malignant cells. The purpose of this study was to explore the genetic diversity of human leukocyte antigen (HLA)-A and HLA-B in patients with PCa and healthy controls (HCs) by performing HLA genotyping using NGS technology. The analysis highlighted statistically significant differences (*p* < 0.05) in the prevalence of three alleles (A*11:01, A*24:02, and B*18:01). Among the HCs analyzed, 14.89% had A*11:01, 20.21% had A*24:02, and 30.61% had B*18:01; while 5.21% of patients with PCa presented A*11:01, 9.38% presented A*24:02, 18.08% presented B*18:01. Odds ratio (OR) calculations underlined a negative association between the three alleles and the risk of PCa (OR < 1). The results presented in this study suggest a protective role of A*11:01, A*24:02, and B*18:01 in PCa.

## 1. Introduction

Prostate cancer (PCa) is one of the most common malignant tumors among men worldwide, with a heterogeneous clinical presentation. Individuals diagnosed with localized prostate cancer falling within a low to intermediate risk of relapse generally experience an encouraging prognosis. The typical diagnostic approach for prostate cancer involves a prostate biopsy, usually prompted by assessments of prostate-specific antigen levels in the blood or digital rectal examinations.

Integrating insights from different studies reveals a multifaceted understanding of prostate cancer susceptibility, progression, and potential therapeutic interventions. A significant association is revealed between family history and prostate cancer susceptibility, particularly revolving around the HOXB13 G84E variant located in the 17q21-22 region, denoting a critical predisposition factor [1]. This variant, consequential in prostate development, has a notably greater prevalence in early-onset, familial prostate cancer cases, shedding light on hereditary implications and potential risk assessment strategies.

In addressing the broader diagnostic landscape, it is crucial to acknowledge that most new diagnoses are low-grade tumors, with a subset progressing to advanced, potentially lethal, prostate cancer.

Moreover, the role of the prostate-specific homeoprotein NKX3.1 is highlighted as a suppressor of prostate cancer initiation through the protection of mitochondria from oxidative stress, thereby revealing a nonnuclear function pivotal in suppressing prostate cancer [2]. Analysis of its expression and localization may contribute significantly to risk assessment, particularly for those under active surveillance, potentially aligning with a precision prevention paradigm.

Additionally, further investigations have identified a set of androgen-responsive genes such as transglutaminase 4 (TGM4) that could serve as potential tumor-associated antigens (TAAs) for prostate cancer [3]. This antigen, found to be highly expressed in prostate tumors and correlating with unfavorable prognosis, has demonstrated immunogenicity and poses as a potential immunotherapy target.

The exploration of such antigens can be pivotal in developing novel immunotherapeutic strategies, thereby addressing the persistent need for more innovative and effective treatments in advanced prostate cancer cases.

Curative therapeutic strategies for such localized conditions encompass radical prostatectomy or ablative radiation therapy [4]. Following prostatectomy, if there’s a recurrence of the disease, salvage radiotherapy combined with androgen deprivation therapy is recommended for local relapse, whereas for systemic relapse, a regimen combining androgen deprivation therapy with chemotherapy or innovative agents targeting androgen signaling is preferred [5]. In advanced stages, prostate cancer can exhibit resistance to androgen depletion, subsequently reaching a castration-resistant phase deemed untreatable. The treatments in contemporary use for prostate cancer do present considerable side effects, leading contemporary research to pivot towards novel modalities like leveraging genetic biomarkers for precise gene therapy and utilizing nanotechnology for specific, controlled interventions [6]. The genotypic and immunologic characteristics of PCa hinder the effective infiltration of immune cells into the neoplastic tissue environment. Because of these limiting factors, the host immune system is compromised in its ability to generate a robust antitumor response, thus failing to effectively mitigate or eradicate the neoplasm. Indeed, PCa is labeled as a “cold tumor”, and is characterized by the low presence of neoantigens, reduced MHC expression, limited infiltration of T cells, and low responsiveness to immunotherapy [7,8,9]. The players responsible for the activation of T cell-mediated immune responses against cancer cells are encoded by human leukocyte antigen (HLA) genes, which are located within the most polymorphic regions of the human genome. The elevated degree of polymorphism and heterozygosity provides the immune system a selective edge when faced with the vast array of microorganisms and antigens encountered by the host [10]. HLA class I and II molecules are located on the surface of nucleated cells and antigen-presenting cells (APCs), respectively, and the specific recognition of peptide-HLA complexes is mediated by T-cell receptors (TCR) on CD8+ and CD4+ cells [11,12]. Moreover, CD8+ activation can promote cancer regression by recognizing antigen-MHC I complexes [13]. Despite a diversified range of immune effector mechanisms showing the potential to inflict harm upon tumors, the primary significance resides in the orchestrated actions of CD8 T cells. It has been documented that there is a correlation between the presence of activated CD8 T cells in neoplastic tissues and an increase in patient survival rates [14]. Furthermore, the realm of adoptive immunotherapy has unveiled promising prospects, wherein engineered T cells expressing receptors derived from tumor-reactive CD8 T cells demonstrate the potential for neoplasm regression [15,16]. Another therapeutic intervention involves incorporating cutting-edge strategies purposed to alter the host’s immune response against cancer cells. The adoption of immune checkpoint inhibitors (ICIs) [17] emerges as an innovative technique in this field. ICIs, including CTLA-4 and PD-1, are pivotal in maintaining immune homeostasis and self-tolerance, serving as integral components of the intricate network regulating immune responses. These checkpoints are crucial in cancer immunotherapy as tumors can exploit inhibitory mechanisms to evade immune attack. Molecules such as Ipilimumab, Nivolumab, Pembrolizumab, and Avelumab have been developed to block the activity of these checkpoints and augment the immune response against cancer cells. These therapies aim to disrupt inhibitory interactions between T cells and tumor cells, promoting T cell activation and proliferation. The goal is to stimulate the immune system to selectively recognize and target tumor cells while maintaining immune balance and preventing autoimmunity [18,19].

Characterization of HLA loci and identification of their association with human diseases such as cancer represent a critical avenue for precision and personalized medicine. Malignant cells possess the capacity to downregulate or altogether abrogate MHC I-mediated antigen presentation. Activation of an immune response mechanism ensues when a T lymphocyte identifies a peptide on MHC Class I as foreign, leading to the proliferation of these lymphocytes and subsequent destruction of the targeted cell [20]. A prevalent strategy employed by many tumor cells to evade immune system detection involves the reduction in or complete absence of MHC Class I expression. The diminished presence of these MHCs on their surface renders tumor cells less detectable to T lymphocytes, allowing their evasion from destruction. Several factors can underpin the lack of MHC Class I expression in tumor cells. These encompass issues related to the synthesis or transport of HLA, challenges in antigen processing, or the absence of essential accessory proteins [21]. Preliminary data indicates an epigenetic silencing of MHC Class I genes in prostate cancer (PCa), signifying that those alterations at the DNA or RNA level hinder MHC Class I expression without modifying the actual DNA sequence [22]. Further, research indicates that radiation therapy can elevate MHC Class I expression in tumor cells, facilitating the presentation of distinct peptides identifiable by the immune system [23]. Elevated MHC Class I levels on a tumor could potentiate the activation and proliferation of CD8+ T lymphocytes in the tumor, invoking a heightened immune response. Nevertheless, despite the potential benefits of enhanced MHC Class I expression in augmenting the immune response against tumors, it may be inadequate on its own, especially if tumors exhibit other immunosuppressive traits. This MHC modulation serves to attenuate or completely obfuscate their immunogenicity to CD8+ T lymphocytes, without detrimentally impacting their proclivity for proliferation and metastatic dissemination [24,25,26,27,28]. To date, several studies have investigated alterations in MHC I expression patterns in PCa and the association of HLA loci with clinical outcome and prognosis. Specific allelic variations within critical immune system genes, notably HLA-A02:01 and HLA-A11, have been positively correlated with the progression of prostate cancer following radical prostatectomy. This suggests that individuals harboring these particular genetic polymorphisms are at an elevated risk of experiencing post-surgical advancement of their prostate malignancy. Consequently, distinct immunogenetic profiles may serve as prognostic markers, offering a nuanced understanding of disease trajectory in prostate cancer [29,30,31]. In the present study, we applied NGS Illumina technology to genotype two HLA class I loci (HLA-A and HLA-B) in patients with PCa and healthy controls (HCs).

## 2. Results

### Allele Frequencies of HLA-A and HLA-B in Patients with PCa and HCs

Table 1 shows the allele frequencies and differences found for the two HLA class I loci (HLA-A and HLA-B) in patients with PCa and HCs. Within HLA-A, A*11:01 was mostly present in the HC population with a frequency of 14.89%, compared to 5.21% in the PCa population (OR:0.314, *p* = 0.026). The A*24:02 frequency was higher in the HC population (20.21%) than that in the PCa population (9.38%) (OR:0.408, *p* = 0.0414). Furthermore, B*18:01 showed a frequency of 30.61% in the HC population compared to 18.08% in the PCa population (OR:0.500, *p* = 0.046). In regard to the remaining alleles detected by HLA genotyping in both populations, no statistically significant difference was detected.

## 3. Discussion

MHC molecules, specifically class I and II, play pivotal roles in adaptive immunity. MHC class I molecules are crucial for presenting endogenous antigens, typically derived from intracellular pathogens, or transformed cells, to CD8+ T cells. MHC class II, on the other hand, primarily presents exogenous antigens to CD4+ T cells. Both are key players in orchestrating immune responses against a myriad of threats, including neoplastic cells. In the context of oncology, the interaction between cancer cells and the immune system has emerged as a significant research area. In particular, ICIs have gained substantial attention in PCa research, given their role in modulating immune responses and their potential as therapeutic targets. It is now understood that some cancer cells, including PCa cells, can employ various mechanisms to escape immune surveillance. A primary strategy is downregulating MHC expression through epigenetic modifications, thereby diminishing the potential of immune cells to recognize and combat them. This poses a significant hurdle in developing effective immunotherapies, given that T cell recognition is largely predicated on the ability to engage with these MHC molecules [31,32]. This study examined the genetic diversity of two MHC class I loci (HLA-A and HLA-B) in patients with PCa and a control population using NGS. Despite the small size of the populations analyzed, the results provide new information about the genetic makeup of these genes in PCa. Statistical analysis of MHC class I data led us to identify three alleles associated with PCa risk. Both A*24:02 and A*11:01 displayed higher frequencies in HCs than in patients with PCa. Previous studies reported an association between A*24:02 and PCa. In a study by Stokidis et al., A*24:02-positive patients showed a more favorable clinical outcome and slower cancer progression [9]. In addition, another study reported increased overall survival and stronger immune responses following a HER-2/neu hybrid polypeptide vaccine in A*24-positive patients [33], corroborating the protective role played by A*24:02 in PCa. A*24:02 presence may increase the immunogenicity of cancer cells by enhancing immunosurveillance of the tumor area and eliciting cytotoxic T lymphocyte responses. Regarding the effects of A*11:01 in cancer patients, the evidence remains controversial. The protective effects of this allele were confirmed in a study where none of the patients with skin cancer presented A*11:01 [34]. In contrast, the frequency of A*11:01 patients with stable and progressive lung cancer was higher than that of the control group [35]. Finally, the frequency of B*18:01 was higher in the HC group than that in the PCa group. B*18:01 was recently associated with the risk of subacute thyroiditis (SAT) recurrence [36,37].

However, to our knowledge, this is the first time this allele has been linked to cancer risk. It is important to emphasize a limitation of our study. Due to the relatively small size of the samples examined, further analyses on a larger population are essential to validate our findings and delve deeper into their implications. Another constraint in our study was the absence of comprehensive clinical data for each participant, which was unfortunately unavailable during the execution of this study. Further investigation is needed to enhance our understanding of the protective roles of A24:02, A11:01, and B*18:01 in PCa.

It is crucial to note the age group disparities in our sample. This arose from challenges in recruiting participants over 65 who did not have significant health issues. As a result, we opted to compare our target population with a relatively younger cohort devoid of pre-existing conditions that might skew our outcomes. Still, age remains a factor that could introduce variables into our results. In the near future, we aim to compare with an age group closer to our own.

## 4. Material and Methods

### 4.1. Sample Collection and PBMCs Isolation

Fifty patients diagnosed with PCa and fifty male healthy controls (HCs) were registered for this study (Table 2) between November 2018 and January 2020.

PCa patients who underwent biopsies at the Urology Unit of the University Hospital of Sassari, and HCs at the Transfusion Center of AOU, Sassari, were enrolled in this study. Peripheral whole blood samples were collected in K^+^-EDTA test tubes from both PCa and HCs individuals. Ficoll–Histopaque gradient centrifugation (Sigma-Aldrich, St. Louis, MO, USA) was used to separate peripheral blood mononuclear cells (PBMCs) from the other blood components. PBMCs were stored at −80 °C in fetal bovine serum and dimethyl-sulfoxide (DMSO) (Sigma-Aldrich, St. Louis, MO, USA) until further use.

### 4.2. Genomic DNA Extraction

PBMCs were washed twice in Phosphate Buffer Saline 1X (PBS) and resuspended in 200 µL PBS 1X. Genomic DNA extraction was performed using a DNeasy Blood and Tissue Kit, following the manufacturer’s instructions (Qiagen, Germantown, MD, USA). The final DNA concentration was measured using Nanodrop One (Thermo Scientific, Waltham, MA, USA), and the DNA quality was assessed by determining the following absorbance ratios: A260/A280 and A260/A230.

### 4.3. Library Preparation and HLA Genotyping

Both patients with PCa and HCs were genotyped for HLA-A and HLA-B using an CRS4-NGSC in-house protocol. A modified long-range PCR protocol was applied to amplify the entire gene region from the 5’UTR to the 3’UTR of HLA-A and HLA-B loci [38]. Primers for HLA-A were redesigned, and amplifications were performed using duplex PCR (two loci for each reaction). A Qubit fluorimeter was used to quantify the PCR products, which were pooled in equimolar quantities. Libraries were obtained using Nextera DNA Flex with 100 ng of DNA and indexed with IDT for Illumina Nextera DNA UD Indexes Primer Set (Illumina, San Diego, CA, USA). After purification of the PCR products with 1X AMPure XP beads (Beckman Coulter, Brea, CA, USA), the libraries were quantified using a Qubit fluorimeter. A loading pool consisting of 96 samples was diluted to 9 pM before sequencing using a MiSeq Reagent Kit v3 600-cycle (Illumina, San Diego, CA, USA).

### 4.4. Bioinformatic and Statistical Analysis

Demultiplexing and FASTQ file generation were conducted on a BaseSpace Sequence Hub (Illumina, San Diego, CA, USA). HLA typing data were analyzed using NGSengine software 2.31.0 (GenDX, IL, USA) and manual data review. For each locus, “not determined” data were removed from the count. The allele frequencies were calculated using the direct counting method, and the differences between patients with PCa and HCs were calculated using a two-sided Fisher’s exact test. A *p* < 0.05 was considered statistically significant. GraphPad Prism 8.2.0 software (GraphPad Software, San Diego, CA, USA) was used for the statistical analysis.

## 5. Conclusions

By examining a larger and more diverse patient population, we intend to reinforce the robustness of our findings and potentially pinpoint other critical alleles. We propose in-depth molecular studies to uncover the precise roles of A24:02, A11:01, and B*18:01 in PCa. This encompasses understanding how these alleles might shape the immune response, especially within the tumor microenvironment. With the advent of personalized medicine, understanding a patient’s genetic makeup becomes paramount. We foresee delving deeper into therapeutic strategies based on the presence or absence of these alleles, potentially leading to more effective and targeted treatments.

Additionally, in the future, we plan to assess the immune cell populations in the primary prostate cancer tumors to determine or indicate a potential functional and/or phenotypic impact of these MHC alterations on the tissue sections.

In conclusion, we advocate for a broad collaborative research effort to validate our findings, focusing on molecular interactions, biochemical pathways, and the implicated immune responses.

## Figures and Tables

**Table 1:** The frequencies and ORs of the HLA-A and HLA-B alleles were estimated between PCa patients and HCs.

Allele	PCa 2n (F%)	HCs 2n (F%)	OR	*p*-Value
A*01:01	3 (3.1%)	3 (3.2%)	0.978	ns
A*01:02	/	1 (1.06%)	0.000	ns
A*02:01	16 (16.66%)	15 (15.96%)	1.053	ns
A*02:05	13 (13.54%)	8 (8.51%)	1.684	ns
A*02:06	1 (1.04%)	/	0.000	ns
A*02:09	1 (1.04%)	/	0.000	ns
A*03:01	6 (6.25%)	2 (2.13%)	3.067	ns
A*11:01	5 (5.21%)	14 (14.89%)	0.314	**0.026**
A*23:01	1 (1.04%)	2 (2.12%)	0.484	ns
A*24:02	9 (9.38%)	19 (20.21%)	0.408	**0.0414**
A*24:03	/	1 (1.06%)	0.000	ns
A*25:01	/	1 (1.06%)	0.000	ns
A*26:01	6 (6.25%)	3 (3.2%)	2.022	ns
A*29:01	1 (1.04%)	2 (2.13%)	0.484	ns
A*29:02	2 (2.08%)	1 (1.06%)	1.979	ns
A*30:01	3 (3.12%)	3 (3.2%)	0.978	ns
A*30:02	12 (12.5%)	12 (12.76%)	0.976	ns
A*30:04	1 (1.04%)	/	0.000	ns
A*31:01	1 (1.04%)	/	0.000	ns
A*32:01	9 (9.38%)	5 (5.32%)	1.841	ns
A*33:01	5 (5.21%)	2 (2.13%)	2.527	ns
A*68:01	1 (1.04%)	/	0.000	ns
A* ND	4	6		
B*07:02	5 (5.32%)	2 (2.04%)	2.697	ns
B*07:05	2 (2.13%)	2 (2.04%)	1.043	ns
B*08:01	2 (2.13%)	2 (2.04%)	1.043	ns
B*13:02	3 (3.19%)	3 (3.06%)	1.044	ns
B*14:02	5 (5.32%)	4 (4.08%)	1.320	ns
B*15:01	4 (4.25%)	1 (1.02%)	4.311	ns
B*15:17	1 (1.06%)	2 (2.04%)	0.516	ns
B*15:18	1 (1.06%)	1 (1.02%)	1.043	ns
B*18:01	17 (18.08%)	30 (30.61%)	0.500	**0.046**
B*18:03	/	1 (1.02%)	0.000	ns
B*27:05	/	1 (1.02%)	0.000	ns
B*35:01	9 (9.57%)	9 (9.18%)	1.047	ns
B*35:02	3 (3.19%)	4 (4.08%)	0.775	ns
B*35:03	/	2 (2.04%)	0.000	ns
B*35:08	1 (1.06%)	/	0.000	ns
B*38:01	5 (5.32%)	3 (3.06%)	1.779	ns
B*39:06	1 (1.06%)	/	0.000	ns
B*40:02	1 (1.06%)	/	0.000	ns
B*40:06	/	1 (1.02%)	0.000	ns
B*44:02	2 (2.13%)	2 (2.04%)	1.043	ns
B*44:03	2 (2.13%)	1 (1.02%)	2.132	ns
B*44:05	2 (2.13%)	/	0.000	ns
B*45:01	1 (1.06%)	1 (1.02%)	1.043	ns
B*47:01	1 (1.06%)	1 (1.02%)	1.043	ns
B*49:01	4 (4.25%)	4 (4.08%)	1.044	ns
B*51:01	3 (3.19%)	4 (4.08%)	0.775	ns
B*51:08	/	1	0.000	/
B*52:01	3 (3.19%)	3 (3.06%)	1.044	ns
B*53:01	1 (1.06%)	1 (1.02%)	1.043	ns
B*55:01	3 (3.19%)	2 (2.04%)	1.582	ns
B*56:01	1 (1.06%)	/	0.000	/
B*57:01	1 (1.06%)	/	0.000	/
B*58:01	9 (9.57%)	8 (8.16%)	1.191	ns
B*58:22	/	1 (1.02%)	0.000	ns
B*73:01	1 (1.06%)	1 (1.02%)	1.043	ns
B* ND	6	2		

Abbreviations: ND, not determined and ns means that the *p* value is higher than 0.05 (*p* > 0.05). Statistically significant differences are displayed in bold.

**Table 2:** Demographic and clinical information about PCa patients and HCs.

	PCa (*n* = 50)	HCs (*n* = 50)
Age (mean ± SD)	70.7 ± 8.1	58.4 ± 7
Serum PSA		
≤4 ng/mL	7	
>4 ng/mL	43	
Gleason Score (GS)		
GS = 6	23	
GS = 7	16	
GS ≥ 8	9	
Unknown	2	

## Data Availability

Not applicable.
